# Investigating allergic rhinitis effects on laryngopharyngeal reflux in Sudanese people during the Sudanese armed conflict

**DOI:** 10.1038/s41598-025-07517-1

**Published:** 2025-07-02

**Authors:** Ahmed Ali, Mohamed H. Elbadawi, Mohammed Aldirdiri, Mohammed Abdalgader, Nusaiba Hassan, Filza Khan, Fatima Hussein, Rayan Hamid, Mifrah Sherwani, Leina Elomeiri, Shahad Elbadawi, Saba El-Bashir, Azza Mohammed, Abdelrahman Idris, Eibaa Syed, Leina Osman, Alaa Hussien, Esra Eltayeb

**Affiliations:** 1 Faculty of Medicine, University of Khartoum, Khartoum, Sudan; 2 East Lancashire Hospitals NHS Trust, Blackburn, UK; 3 Omdurman Islamic University, Omdurman, Sudan; 4 Karachi Medical and Dental College, Karachi, Pakistan; 5 Faculty of Medicine, Alzaiem Alazhari University, Khartoum, Sudan; 6 Faculty of Medicine, University of Kassala, Kassala, Sudan

**Keywords:** Immunology, Respiratory signs and symptoms

## Abstract

Laryngopharyngeal reflux (LPR) involves the reflux of stomach contents into the upper aerodigestive tract and is often linked to allergic rhinitis (AR), an inflammatory nasal condition. This study investigates the potential association between AR and LPR in Sudanese adults, focusing on individuals with asthma. Analytical, community based cross-sectional study was conducted online (June 11–July 21, 2024) using convenient sampling, which included Sudanese adults aged 18 and older. Validated Arabic versions of the Reflux Symptom Index (RSI) and the Score for Allergic Rhinitis (SFAR) assessed LPR and AR, respectively. Data analysis using SPSS version 26 utilized statistical tests, including univariate analysis and logistic regression, to explore associations. Among our 1,195 participants, a strong positive correlation was found between RSI and SFAR scores (*r* = 0.595, *p* < 0.001). Participants with AR were six times more likely to have LPR symptoms (OR = 5.956, *p* < 0.001). Females and younger adults reported higher rates of both conditions, with family history of allergies, nasal symptoms, and environmental allergen exposure significantly associated with LPR. Asthma, though present in a small subset, did not significantly alter the AR-LPR association. The study highlights a strong association between AR and LPR, particularly in females and younger adults, emphasizing the need for integrated management. Future research should explore asthma’s role in larger cohorts and utilize objective diagnostic tools to establish causality. The findings also stress the importance of increased funding for biomedical research in resource-limited settings like Sudan.

**Supplementary Information:**

The online version contains supplementary material available at 10.1038/s41598-025-07517-1.

## Introduction

Laryngopharyngeal reflux (LPR) is the reflux of stomach contents into the upper aerodigestive system without regurgitation or heartburn^1^. Heartburn and acid regurgitation, especially at night and when lying down, are indications of Gastroesophageal reflux disease^2^. An endoscopy or pH monitoring can confirm a diagnosis of esophagitis^2^. The diagnosis of LPR is more intricate and requires both therapeutic strategies and in-depth clinical examination^1,3^.

Because of its perplexing symptoms, such as hoarseness in voice, throat clearing, and globus pharyngeus, it is difficult to estimate the prevalence of LPR^1,2^. There are differences between LPR and Gastroesophageal reflux disease (GERD) in terms of diagnosis, therapy, and psychological effects^4^. Furthermore, the mechanism of LPR is not exclusive to the gastrointestinal tract. Its symptoms can be caused by various factors, including vocal abnormalities, medications, neurogenic conditions, pulmonary issues, smoking, environmental factors, and allergies^2^.

According to guidelines, people with typical GERD symptoms and no alarm characteristics may benefit from an empirical proton pump inhibitor (PPI) trial^2^. While an empirical trial of PPIs is feasible for individuals with concurrent esophageal symptoms, upfront ambulatory reflux monitoring is advised for patients with isolated laryngeal symptoms due to the uneven response to empiric PPI therapy in patients with laryngopharyngitis^5^. It is frustrating that a considerable percentage of patients (30–45%) do not respond well enough to PPI treatment and lifestyle changes^3^. Notably, according to present recommendations, laryngoscopy alone is insufficient for diagnosing LPR. It is also evaluated using a variety of tests, such as oesophageal manometry, pH, and impedance monitoring to detect non-acidic reflux episodes. More recently, oropharyngeal pepsin testing is also being used to diagnose LPR^6^.

One of the most prevalent illnesses in the world, allergic rhinitis (AR) is an inflammatory disease that develops in the nasal mucosa as a result of allergen exposure^7^. Worldwide, the frequency of AR varies from 5 to 22%^8^. Given their similarities, AR and asthma might be thought of as two equivalent airway illnesses. Several other symptoms are associated with AR, such as rhinorrhea, sneezing, nasal irritation, and nasal congestion or obstruction^9^.

A limited amount of research has been done that suggests an informal connection between allergic rhinitis and laryngopharyngeal disease, even though patients with asthma present with both of these diseases. Nonetheless, numerous studies have indicated that AR and LPR may be related to one another despite neither having asthma since they share many symptoms related to aerodigestive tract irritation^8,10^.

Our research aims to explore the potential comorbidity of allergic rhinitis and laryngopharyngeal reflux illness in a general population. We hypothesize that laryngopharyngeal reflux is associated with allergic rhinitis.

## Methodology

### Study design and participants

An analytic, community-based cross-sectional study was conducted in Sudan between June 11 and July 21, 2024. Participants aged 18 years and older, residing in Sudan, and who completed all items of the Reflux Symptom Index (RSI) questionnaire were included. Recruitment occurred online through various social media apps using targeted posts explaining the study’s purpose.

### Sampling

A convenient sampling method was used to collect the data from the community. Data collectors were recruited to send the online form via social media apps. Sample size was calculated using sample size equation for unknown population:

$$s=\frac{{{Z^2} \times P\left( {1 - P} \right)}}{{{e^2}}}$$

s = sample size. Z = Z score for used confidence interval (95%) = 1.96. P = Probability of the disease = 0.5 (unkonwn). e = margin of error = 0.05. By multiplying the design effect (3x sample size) and adding a non-response rate of 10%, the final sample size is 1268.

### Measurements

Data were collected via an online survey, which included demographic information (age, gender, region of residence, income, profession), clinical history items, reflux symptom index (RSI) and score for allergic rhinitis (SFAR). Items to assess the clinical history of the participants included: asthma clinical diagnosis, family history of asthma, history of atopy and their exacerbating factors. Two previously pilotted, validated questionnaires were also administered. The first one was a validated Arabic version of the reflux symptom index (RSI) with cronbach alpha value of 0.72^8,11^, which was employed to assess the likelihood of LPR. The RSI is a self-administered nine-item questionnaire with each item scored on a scale of 0–5, yielding a total score ranging from 0 to 45 points. A cut-off score of 13 or higher suggests possible LPR. The survey also incorporated the Seasonal Allergic Rhinitis (SFAR) tool to evaluate allergic rhinitis symptoms by asking questions about: family history, nose symptoms, eye symptoms and clinical history. SFAR tool aims to determine the prevalence of allergic rhinitis using a cut value of 7^12^ and cronbach alpha of 0.79 (Supplementary file 1). No medical examinations or diagnostic procedures were performed during the study period. The response rate for this survey was 94.2%, based on a total sample size of 1,268 and 1,195 completed responses.

### Data analysis

The data were initially collected using Excel and then cleaned using SPSS version 26. Normality test was performed for continuous data, which are: age, reflux symptoms index scale, changes in residency and score for allergic rhinitis, where the Kolmogorov normality test indicates non-normal distribution (P value was <0.001 for all of them), so non-parametric tests were used. Missing value analysis was performed to estimate the missing percentages in all variables which yielded less than 5% in all variables. Then descriptive statistics and frequency tables were used to describe the data. Mann-Whitney test was used to study the differences between age and changes in residency against the reflux symptom index and allergic rhinitis. Chi-squire and Fisher exact tests were used to study the associations between multiple socio-demographic and clinical factors against the reflux symptom index and allergic rhinitis. Spearman’s rho test was used to evaluate the association between allergic rhinitis score and reflux symptom index score. Chi-squire test was also used to study the differences among allergic rhinitis groups and reflux symptoms. Binomial logistic regression was also used to study the factors affecting allergic rhinitis (binary variable), so socio-demographic characteristics with a p-value less than 0.05 and reflux symptoms were selected to enter the model. In all tests p-value of less than 0.05 was indicated as a significant result.

### Ehical approval and consent of participants

Ethical approval was obtained from the Ethical Committee of the Ministry of Health, River Nile State. This approval covered all aspects of the study, including participant recruitment, data collection, analysis, publication, and data usage. Informed consent was obtained from all participants before their involvement in the study. All procedures adhered to the principles of the Declaration of Helsinki and complied with relevant guidelines and regulations, including the STROBE guidelines for reporting observational studies.

## Results

### Socio-demographic characteristics

A sample of 1195 was collected in this study. The median age was 24 ± 7 years. The majority of the participants (63.9%) were females, and more than two-thirds (75.2%) were single. Most of the participants were from: River Nile state (24.7%), Kassala state (17.5%) and North state (15.1%). Almost (83.8%) had a bachelor’s degree, and only 1 participant reported to be illiterate (0.1%). (42.4%) of the participants were from non-medical fields, while the rest were medical field workers from different specialities. More than two-thirds of the participants (67.9%) didn’t work, and only 348 (10.9%) participants indicated family income was insufficient for their needs (Table 1).

Almost quarter of the participants (278,23.3%) reported having positive reflux symptoms indicating LPR symptoms. There was a significant, very low negative association between age and LPR symptoms index scale (*r* = – 0.90, p-value = 0.002). Females tend to significantly have a higher percentage (26.7%) of LPR symptoms compared to males (17.2%) (p-value<0.001). Moreover, participants who aren’t currently studying in universities reported higher percentages (35.8%) of these symptoms than university students (p-value = 0.042). Marital status, grade, and working status didn’t show any reported significance to LPR (Table 2).

Females tend to significantly report a higher percentage (73.6%) of AR compared to males (26.4%) (p-value < 0.001). Moreover, the highest significant percentage of AR was noticed in medical students (40.5%) (p-value = 0.014). There weren’t any associations between AR and age, marital status, or income (Table 3).

### Clinical factors contributing to laryngopharyngeal reflux symptoms

There was a significant difference between patients with asthma and patients without it regarding laryngopharyngeal reflux symptoms, with the highest percentage of asthmatic patients having no LPR symptoms (53.5%) (p-value = 0.001). Having a family history of allergy in the nose, chest, or skin was significantly associated with a higher percentage of LPR symptoms compared to those with no history (29.9% vs. 13.6%) (p-value < 0.001). The highest reported type of allergy in the family was chest and nose allergy for the father (56.5%), skin and chest allergy for the mother (62.5%), and skin, nose, and chest allergy for both sons/daughters and siblings (57.1% for both) (p-value < 0.01) (Table 4).

There was a significant association between having a runny nose, sneezing, nasal congestion, and having LPR symptoms (p-value < 0.001). Participants who had nose problems during most of the year (all seasons of the year) had a significantly higher percentage of LPR symptoms (67.1%) compared to those who had nose problems in one month or some months of the year (p-value < 0.001). The allergy-exacerbating factors significantly associated with LPR symptoms were dust, insects, animals, and pollen (p-value < 0.001 for all). However, participants without allergies to any of these factors reported a higher percentage of no LPR symptoms. Participants with a history of allergy had a significantly higher percentage (38.9%) of laryngopharyngeal reflux symptoms (p-value < 0.001), especially among ones who had a positive test in skin or blood tests for allergy (46.5%) (p-value < 0.001). Moreover, having a skin, nose, or chest allergy diagnosis led to a significantly higher percentage of reflux symptoms (p-value < 0.001) (Table 5).

### Association between laryngopharyngeal reflux and allergic rhinitis

Participants with allergic rhinitis were found to have significantly six times more laryngopharyngeal reflux symptoms compared to those without rhinitis (OR = 5.956, 95% CI: 4.439–7.992, p-value < 0.001). Among asthmatics, there was no significant difference in LPR symptoms between those with AR and those without (p-value = 0.191). However, in participants without asthma, the significant association between AR and LPR remained present (OR = 5.797, 95% CI: 4.285–7.841, p-value < 0.001).) (Table 6).

The median score of allergic rhinitis (SFAR) was 5 ± 6. While the median of the laryngopharyngeal reflux symptoms index score (RSI) was 5 ± 12. The correlation matrix was formed between the score of allergic rhinitis (SFAR) and the laryngopharyngeal reflux symptoms index score (RSI). There was a significantly high positive association between allergic rhinitis score and laryngopharyngeal reflux symptoms score (*r* = 0.595, p-value<0.001). Moreover, a moderate positive association was also reported between the two scales when dividing the sample into asthmatic and non-asthmatic patients (*r* = 0.50 for asthmatic and 0.59 for non-asthmatic, p-value = 0.001 for asthmatic and < 0.001 for non-asthmatic) (Table 7).

Binomial logistic regression was performed for variables that contribute to allergic rhinitis according to previous univariate analysis. These variables were: gender, faculty, income and laryngopharygeal reflux symptoms. The model has statistically significant results in Omnibus tests (p-value < 0.001) with a Nagelkerke R-squared value of 0.202. Participants with income that is sufficient for basic needs and some additions had significantly 1.6 times less allergic rhinitis compared to those with insufficient income at all (OR = 0.619, C.I = 0.400- 0.956, p-value = 0.031). Moreover, participants with positive laryngopharyngeal reflux symptoms tend to have significantly 5.6 times more allergic rhinitis than ones with negative symptoms (OR = 5.628, C.I = 4.163–7.607, p-value < 0.001) (Table 8).

**Table 1:** The table shows the socio-demographic characteristics of the participants (*N* = 1195).

Variables		Frequency	Percentage %
Age		24^a^	7^b^
Gender	Female	764	63.9%
	Male	431	36.1%
Marital Status	Single	899	75.2%
	Married	190	15.9%
	Engaged	88	7.4%
	Widow	4	0.3%
	Divorced	14	1.2%
Current residency	River Nile	293	24.5%
	Kassala	209	17.5%
	North State	181	15.1%
	Red Sea	145	12.1%
	Khartoum	134	11.2%
	White Nile	74	6.2%
	Gazira	46	3.8%
	Al Qadarif	38	3.2%
	North Kordofan	33	2.8%
	Sennar	25	2.1%
	Blue Nile	10	0.8%
	West Kordofan	2	0.2%
	North Darfur	2	0.2%
	South Darfur	1	0.1%
	South Kordofan	1	0.1%
	East Darfur	1	0.1%
Educational level	Illiterate	1	0.1%
	informal education	1	0.1%
	Primary School	7	0.6%
	Secondary school	127	10.6%
	Bachelor degree	1001	83.8%
	Postgraduates	58	4.9%
University grade	1st year	107	9.0%
	2nd year	141	11.8%
	3rd year	167	14.0%
	4th year	119	10.0%
	5th year	60	5.0%
	6th year	25	2.1%
	Not currently a university student	576	48.2%
Faculty	Medical laboratory sciences	46	3.9%
	Public health	9	0.8%
	Nursing	53	4.5%
	Pharmacy	55	4.6%
	Dentistry	34	2.9%
	Medicine	435	36.6%
	Non-medical fields	504	42.4%
	Not studying in university currently	53	4.5%
Working status	Employed	384	32.1%
	Unemployed	811	67.9%
Family income sufficiency	Not enough at all	130	10.9%
	Sufficient for basic needs only	443	37.1%
	Sufficient for basic needs and some additions	574	48.0%
	Sufficient for way above basic needs and luxuries	48	4.0%

^a^Median.

^b^Interquartile Range.

**Table 2:** Univariate analysis of socio-demographic variables against reflux symptoms (*N* = 1195).

Variables		Laryngopharyngeal reflux symptoms		
		No reflux symptoms (%)	Reflux symptoms (%)	*p*-value
Age		917 (76.7%)^a^	278(23.3%)^a^	0.000^b^**
Gender	Female	560 (73.3%)	204(26.7%)	0.000**
	Male	357 (82.8%)	74(17.2%)	
Marital Status	Single	684 (76.1%)	215(23.9%)	0.283
	Married	152 (80%)	38(20%)	
	Engaged	70 (79.5%)	18(20.5%)	
	Widow	2 (50%)	2(50%)	
	Divorced	9 (64.3%)	5(35.7%)	
Educational level	Illiterate	1 (100%)	0(0%)	0.908
	informal education	1 (100%)	0(0%)	
	Primary School	5 (71.4%)	2(28.6%)	
	Secondary school	95 (74.8%)	32(25.2%)	
	Undergraduates	769 (76.8%)	232(23.2%)	
	Postgraduates	46 (79.3%)	12(20.7%)	
University grade	1st year	78 (72.9%)	29(27.1%)	0.204
	2nd year	101 (71.6%)	40(28.4%)	
	3rd year	124 (74.3%)	43(25.7%)	
	4th year	93 (78.2%)	26(21.8%)	
	5th year	47 (78.3%)	13(21.7%)	
	6th year	16 (64%)	9(36%)	
	not currently a student	458 (79.5%)	118(20.5%)	
Faculty	Medical laboratory sciences	32 (69.6%)	14(30.4%)	0.042*
	Public health	6 (66.7%)	3(33.3%)	
	Nursing	40 (75.5%)	13(24.5%)	
	Pharmacy	48 (87.3%)	7(12.7%)	
	Dentistry	31 (91.2%)	3(8.8%)	
	Medicine	329 (75.6%)	106(24.4%)	
	Non-medical fields	392 (77.8%)	112(22.2%)	
	Not studying in university currently	34 (64.2%)	19(35.8%)	
Working status	Employed	305 (79.4%)	79(20.6%)	0.149
	Unemployed	612 (75.5%)	199(24.5%)	
Family income sufficiency	Not enough at all	93 (71.5%)	37(28.5%)	0.489
	Sufficient for basic needs only	340 (76.7%)	103(23.3%)	
	Sufficient for basic needs and some additions	446 (77.7%)	128(22.3%)	
	Sufficient for way above basic needs and luxuries	38 (79.2%)	10(20.8%)	

^a^Total frequency from the whole population.

*p-value <0.05.

**p-value<0.001.

**Table 3:** Univariate analysis of socio-demographic variables against allergic rhinitis (*N* = 1195).

Variables		Allergic rhinitis		
		Patient without allergic rhinitis	Patient with allergic rhinitis	*p*-value
Age		26.1 ± 6.0^a^	25.9 ± 7.0^a^	0.738
Gender	Female	443(58.9%)	301(73.6%)	0.000**
	Male	309(41.1%)	108(26.4%)	
Marital Status	Single	571(75.9%)	304(74.3%)	0.950
	Married	114(15.2%)	69(16.9%)	
	Engaged	55(7.3%)	30(7.3%)	
	Widow	3(0.4%)	1(0.2%)	
	Divorced	9(1.2%)	5(1.2%)	
Current residency	River Nile	190(25.3%)	99(24.2%)	
	Kassala	134(17.8%)	69(16.9%)	
	North state	111(14.8%)	66(16.1%)	
	Red Sea	89(11.8%)	53(13%)	0.954
	Khartoum	88(11.7%)	39(9.5%)	
	White Nile	42(5.6%)	29(7.1%)	
	Gazira	30(4%)	14(3.4%)	
	Al Qadarif	24(3.2%)	13(3.2%)	
	Kordofan states	23(3.1%)	11(2.7%)	
	Blue Nile	5(0.7%)	4(1%)	
	Darfor states	2(0.3%)	2(0.5%)	
	Sennar	14(1.9%)	10(2.4%)	
Faculty	Medical laboratory sciences	28(3.7%)	17(4.2%)	0.014*
	Public health	4(0.5%)	4(1%)	
	Nursing	27(3.6%)	25(6.1%)	
	Pharmacy	36(4.8%)	18(4.4%)	
	Dentistry	28(3.7%)	4(1%)	
	Medicine	263(35.1%)	165(40.5%)	
	Non-medical fields	334(44.6%)	154(37.8%)	
	Not studying in university currently	29(3.9%)	20(4.9%)	
Educational level	Illiterate	0(0%)	1(0.2%)	0.490
	informal education	1(0.1%)	0(0%)	
	Primary School	5(0.7%)	2(0.5%)	
	Secondary school	77(10.2%)	43(10.5%)	
	Undergraduates	637(84.7%)	338(82.6%)	
	Postgraduates	32(4.3%)	25(6.1%)	
University grade	1st year	74(9.8%)	30(7.3%)	0.824
	2nd year	91(12.1%)	45(11%)	
	3rd year	106(14.1%)	59(14.4%)	
	4th year	72(9.6%)	45(11%)	
	5th year	39(5.2%)	21(5.1%)	
	6th year	16(2.1%)	9(2.2%)	
	Not currently a student	354(47.1%)	200(48.9%)	
Working status	Employed	242(32.2%)	128(31.3%)	0.808
	Unemployed	510(67.8%)	281(68.7%)	
Family income sufficiency	Not enough at all	70(9.3%)	55(13.4%)	0.054
	Sufficient for basic needs only	276(36.7%)	154(37.7%)	
	Sufficient for basic needs and some additions	379(50.4%)	180(44%)	
	Sufficient for way above basic needs and luxuries	27(3.6%)	20(4.9%)	

^a^Median ± interquartile range.

*p-value <0.05.

**p-value<0.001.

**Table 4:** Univariate analysis of clinical history variables against reflux symptoms (*N* = 1195).

Variables		Reflux symptoms		
		No LPR symptoms (%)	LPR symptoms (%)	p-value
Asthma clinical diagnosis	Diagnosed	23(53.5%)	20(46.5%)	0.001*
	Not diagnosed	884(77.5%)	256(22.5%)	
Member of the family with a nose, chest or skin allergy?	Yes	496(70.1%)	212(29.9%)	0.000**
	No	421(86.4%)	66(13.6%)	
Type of father allergy	Doesn’t have allergy	737(79.7%)	188(20.3%)	0.000**
	Nose allergy	94(71.8%)	37(28.2%)	
	Chest allergy	42(61.8%)	26(38.2%)	
	Skin allergy	27(73%)	10(27%)	
	Chest and nose allergy	10(43.5%)	13(56.5%)	
	Skin and chest allergy	2(50%)	2(50%)	
	Nose and skin allergy	4(80%)	1(20%)	
	Skin, nose and chest allergy	1(50%)	1(50%)	
Type of mother allergy	Doesn’t have allergy	684(82%)	150(18%)	0.000**
	Nose allergy	116(70.7%)	48(29.3%)	
	Chest allergy	53(63.9%)	30(36.1%)	
	Skin allergy	32(68.1%)	15(31.9%)	
	Chest and nose allergy	16(47.1%)	18(52.9%)	
	Skin and chest allergy	3(37.5%)	5(62.5%)	
	Nose and skin allergy	11(61.1%)	7(38.9%)	
	Skin, nose and chest allergy	2(28.6%)	5(71.4%)	
Type of son/ daughter allergy	Doesn’t have allergy	836(78.4%)	231(21.6%)	0.006*
	Nose allergy	32(65.3%)	17(34.7%)	
	Chest allergy	22(68.8%)	10(31.3%)	
	Skin allergy	12(66.7%)	6(33.3%)	
	Chest and nose allergy	5(50%)	5(50%)	
	Skin and chest allergy	4(66.7%)	2(33.3%)	
	Nose and skin allergy	3(50%)	3(50%)	
	Skin, nose and chest allergy	3(42.9%)	4(57.1%)	
Type of siblings allergy	Doesn’t have allergy	563(81.4%)	129(18.6%)	0.000*
	Nose allergy	133(74.3%)	46(25.7%)	
	Chest allergy	100(75.8%)	32(24.2%)	
	Skin allergy	37(68.5%)	17(31.5%)	
	Chest and nose allergy	37(63.8%)	21(36.2%)	
	Skin and chest allergy	7(53.8%)	6(46.2%)	
	Nose and skin allergy	24(64.9%)	13(35.1%)	
	Skin, nose and chest allergy	16(53.3%)	14(46.7%)	

*p-value <0.05.

**p-value<0.001.

^a^The participants were allowed to choose more than one option and each option has been analyzed separately.

**Table 5:** Univariate analysis of allergies history against reflux symptoms (*N* = 1195).

Variables		Reflux symptoms		
		No LPR symptoms (%)	LPR symptoms (%)	p-value
Regardless of having cold, did you suffer from any of the following during previous year	Running nose	96(86.5%)	15(13.5%)	0.000**
	Nasal congestion	76(68.5%)	35(31.5%)	
	Sneezing	93(81.6%)	21(18.4%)	
	Running nose and sneezing	49(71%)	20(29%)	
	Running nose and nasal congestion	25(64.1%)	14(35.9%)	
	Sneezing and nasal congestion	36(64.3%)	20(35.7%)	
	All of the above (running nose, sneezing, nasal congestion)	112(46.9%)	127(53.1%)	
	Didn’t have anything	401(94.8%)	22(5.2%)	
Season in which the nose problem occurred?	Didn’t have nose problem	425(94.2%)	26(5.8%)	0.000**
	Winter season	167(75.6%)	54(24.4%)	
	Summer season	166(74.1%)	58(25.9%)	
	Autumn season	52(71.2%)	21(28.8%)	
	Both Autumn and Winter	24(48%)	26(52%)	
	Summer and Winter	40(58%)	29(42%)	
	Summer and Autumn	20(54.1%)	17(45.9%)	
	All seasons	23(32.9%)	47(67.1%)	
Exacerbating Factors of allergy^a^	Dust	648(72.3%)	248(27.7%)	0.000**
	Cold	8(66.7%)	4(33.3%)	0.489
	Perfumes	10(62.5%)	6(37.5%)	0.228
	Insects	90(62.5%)	54(37.5%)	0.000**
	Change in weather	16(80%)	4(20%)	1.000
	Animals	43(58.1%)	31(41.9%)	0.000**
	Pollen	96(62.7%)	57(37.3%)	0.000**
Believing in having an allergy	Yes	385(66.7%)	192(33.3%)	0.000**
	No	532(86.1%)	86(13.9%)	
Taking skin or blood tests for allergy	Yes	80(61.1%)	51(38.9%)	0.000**
	No	837(78.7%)	227(21.3%)	
Allergy test result	I didn’t have the test	835(78.7%)	226(21.3%)	0.000**
	Negative, there is no allergy	28(84.8%)	5(15.2%)	
	Positive, there is allergy	54(53.5%)	47(46.5%)	
Diagnosis of skin, nose or chest allergy	Yes	219(62.8%)	130(37.2%)	0.000**
	No	698(82.5%)	148(17.5%)	

*p-value <0.05.

**p-value<0.001.

**Table 6:** The table shows the univariate analysis between allergic rhinitis and reflux symptoms (*N* = 1195).

Allergic rhinitis	Reflux symptoms					
	No LPR symptoms		LPR symptoms		Odd ratio (C.I^a^)	p-value
	Count	Column N %	Count	Column N %		
SFAR score						
Patient without allergic rhinitis	662	74.5%	90	33.0%	5.956 (4.439–7.992)	0.000**
Patient with allergic rhinitis	226	25.5%	183	67.0%		
SFAR score in patients with asthma						
Patient without allergic rhinitis	5	22.7%	1	5.3%	5.294 (0.560-50.080)	0.191
Patient with allergic rhinitis	17	77.3%	18	94.7%		
SFAR score in patients without asthma						
Patient without allergic rhinitis	652	76.0%	89	35.3%	5.797 (4.285–7841)	0.000**
Patient with allergic rhinitis	206	24.0%	163	64.7%		

^a^Confidence interval.

*p-value <0.05.

**p-value<0.001.

**Table 7:** The table shows the correlation matrix between allergic rhinitis scale(SFAR) and laryngopharyngeal reflux scale (RSI) (*N* = 1195).

Variable	Median	IQR	Correlation	
			*r* value	*p*-value
All patients				
The score for allergic rhinitis	5	6	0.595	0.000**
Laryngopharyngeal reflux symptom index score	5	12		
Patients with asthma				
The score for allergic rhinitis	10	5.5	0.501	0.001*
Laryngopharyngeal reflux symptom index score	9	16		
Patients without asthma				
The score for allergic rhinitis	4	5	0.591	0.000**
Laryngopharyngeal reflux symptom index score	4	11		

*p-value <0.05.

**p-value<0.001.

**Table 8:** The table shows binomial logistic regression for variables that contribute to allergic rhinitis (*N* = 1195).

Variables	B	S.E.	Wald	df	*p*-value	Odd ratio	95% C.I^b^ for odd ratio	
							Lower	Upper
Gender(ref = female)								
Male	− 0.512	0.150	11.687	1	0.001*	0.599	0.447	0.804
Your faculty (ref = Medical laboratory sciences)			8.940	7	0.257			
Public health	0.412	0.845	0.238	1	0.626	1.510	0.288	7.905
Not studying in university currently	0.162	0.466	0.122	1	0.727	1.176	0.472	2.930
Nursing	0.523	0.448	1.358	1	0.244	1.687	0.700	4.062
Pharmacy	0.234	0.457	0.262	1	0.609	1.263	0.516	3.092
Dentistry	-1.051	0.650	2.612	1	0.106	0.350	0.098	1.250
Medicine	0.196	0.351	0.312	1	0.576	1.217	0.611	2.422
Non-medical fields	− 0.030	0.352	0.007	1	0.931	0.970	0.487	1.934
Is family income sufficient for your needs? (ref = not enough at all)			6.278	3	0.099			
Sufficient for basic needs only	− 0.272	0.227	1.430	1	0.232	0.762	0.488	1.190
Sufficient for basic needs and some additions	− 0.480	0.222	4.672	1	0.031*	0.619	0.400	0.956
Sufficient for way above basic needs and luxuries	− 0.023	0.375	0.004	1	0.951	0.977	0.469	2.038
Presence of laryngopharyngeal reflux symptoms (ref = NO LPR symptoms)								
Positive LPR symptoms	1.728	0.154	126.192	1	0.000**	5.628	4.163	7.607
Constant	− 0.647	0.393	2.710	1	0.100	0.524		

^b^C.I is confidence interval.

*p-value <0.05.

**p-value<0.001.

## Discussion

Understanding the complex relationships between allergic rhinitis (AR), laryngopharyngeal reflux (LPR), and asthma is crucial given their high prevalence and impact on quality of life^5,13,14^. Moreover, patients with LPR often present with symptoms that overlap with AR and asthma^15^, such as chronic cough, throat clearing, and hoarseness of voice, which further hinders precise diagnoses and proper management. We investigated these conditions’ reciprocal relations to better understand their prevalence, associations, and implications, with the primary focus on elucidating the connections between them and exploring potential solutions for management.

The innovative aspect of our study lies in its comprehensive analysis of how AR and LPR intersect, particularly within a young and educated population. With a sample size of 1,195 participants, our study benefits from enhanced statistical power, allowing for more reliable and generalizable results. This large sample size also increases the precision of our estimates and strengthens the validity of the observed associations.

In patients with AR, an increased frequency of swallowing is typically observed, often attributed to throat irritation and posterior nasal drip. This heightened swallowing may also be exacerbated by gastroesophageal reflux. The impact of AR on the nasal mucosa may similarly affect the laryngeal mucosa, resulting in congestion, edema, and excessive mucous secretion, which contribute to the symptoms of LPR^10^. This shared effect on the mucosal tissues may elucidate the observed association between LPR and AR.

Additionally, GERD and AR are recognized as significant contributors to chronic cough^16^. Another phenomenon that may explain this association is the identification of Helicobacter pylori within the sinonasal mucosa, despite its primary association with the gastric mucosa, where it is known to promote and contribute to GERD^17^. Eosinophils, a critical component of allergic inflammation, have also been identified in the esophageal mucosa of patients with GERD^18^.

The biological mechanisms underlying the association between AR and LPR are multifaceted. A central aspect of this relationship is the concept of shared inflammatory pathways. These pathways activate group 2 innate lymphoid cells (ILC2s), which rapidly produce type 2 cytokines (IL-5, IL-13, and IL-4) in situ. Consequently, ILC2s play a critical role in the initiation and maintenance of type 2 adaptive immune responses, which ultimately lead to immunoglobulin E (IgE) class switching and mucosal inflammation^7^. This inflammatory environment may extend beyond the nasal cavity to the laryngopharynx, thereby increasing tissue susceptibility to damage induced by reflux. Conversely, in gaseous reflux, pepsin comes into contact with the upper aerodigestive tract (UAT) mucosa, potentially extending as far as the middle ear, and may inflict damage through subsequent episodes of acid reflux^19^. This bidirectional relationship establishes a potential vicious cycle in which each condition may exacerbate the other.

The educational background of our sample was notably high, with 83.8% holding a bachelor’s degree. This demographic profile is reflective of a young, educated population, which may influence the reported rates of LPR, AR, and associated factors. Females in our research reported a significantly higher percentage of both AR and LPR symptoms compared to males (*p* < 0.001). Although Pinart et al. found no sex-related difference in AR prevalence among adults, they did observe a female predominance in adolescents^20^, which may explain the similar trend in our younger sample. The increased prevalence of LPR among females aligns with findings by Liu et al.^21^. Similarly, Lechien et al. reported that females are more likely than males to experience severe LPR symptoms and related voice disorders^22^. These findings might be linked to the hormonal differences between males and females. Research shows that estrogens in females can boost humoral responses, autoimmunity, mast cell reactivity, and delayed type IV allergic reactions, while androgens, progesterone, and glucocorticoids may actually dampen the immune response. Essentially, these immune mechanisms can worsen allergies for females. Plus, T-cell responses fluctuate with the menstrual cycle, which hints at how allergic symptoms can change during that time, as well as during pregnancy, while on oral contraceptives, or with hormone replacement therapy^23^. There are also anatomical differences, like increased laryngeal sensitivity in females, alongside behavioral factors such as how often they seek healthcare, and all of this adds to the disparities.

Notably, our data revealed that younger adults reported significantly higher rates of LPR symptoms than older adults, in contrast to findings by Lechien et al. and others^21,22^, who observed a positive correlation between LPR incidence and age. This discrepancy may be due to the demographic profile of our sample, which consists predominantly of younger adults. Moreover, socioeconomic factors were found to influence AR prevalence, with participants reporting sufficient income showing a reduced likelihood of developing AR. Perry et al. suggested that lifestyle and environmental exposures associated with lower income may increase the risk of developing asthma and allergy^24^.

Overall, a strong association (*r* = 0.595) between AR and LPR was observed. Participants with AR were six times more likely to experience LPR symptoms compared to those without AR (OR = 5.956), an association notably higher than that reported in some previous research^8,9^, likely due to the greater sample size of our study. This finding is also consistent with previous results that have linked having LPR or AR to an increased risk of having the other due to the inflammatory processes shared between these conditions^10,25^, and it further reinforces the need for integrated management strategies for patients presenting with both conditions. However, similar to findings from Kakaje et al.^8^, the presence of AR did not significantly alter the prevalence of LPR symptoms among asthmatic patients, suggesting that asthma may modulate the relationship between rhinitis and LPR, potentially through overlapping or competing mechanisms.

A strong association between a family history of allergies - particularly in the nose, chest, and skin - and the prevalence of LPR symptoms was identified. This suggests that genetic predisposition to allergic conditions may increase susceptibility to LPR, likely due to the inflammatory effects of allergic conditions on the upper airway and potential impact on esophageal function. Environmental allergens such as dust, insects, animals, and pollen were significantly associated with LPR symptoms, emphasizing the role of external triggers in the manifestation of these symptoms. This finding was more solidified by the participants without allergies to these triggers reporting fewer LPR symptoms.

Nasal symptoms such as a runny nose, sneezing, and nasal congestion were significantly linked to LPR symptoms, with those experiencing these symptoms year-round showing a higher prevalence of LPR (67.1%). Similarly, Luk and DelGaudio highlight that chronic rhinosinusitis is associated with an increased risk of LPR^26^, while Lechien et al. concluded that LPR contributes to the development of recalcitrant chronic rhinosinusitis^25^.

The majority of asthmatic patients in our study reported an absence of LPR symptoms (53.5%). This finding contrasts with previous studies that have linked asthma to an increased risk of LPR^8,27^. However, it is important to note that our sample included only 43 subjects diagnosed with asthma (3.5%), which may have limited our ability to detect a significant association between asthma and LPR.

The clinical implications of what we’ve found really highlight the need for integrated care pathways for AR and LPR, especially among women and younger adults. Clinicians ought to use a dual screening approach, tap into environmental modification strategies, and tackle those socioeconomic barriers that often hinder treatment adherence. And when it comes to places like Sudan, where conflict affects everything, focusing on low-cost interventions and community education can really help ease the added burden of these conditions.

### Strengths and limitations

This study has several strengths; it included a large and diverse group of participants, as it was conducted outside of a clinical setting and not limited to those who are severely affected. This enhances the study’s external validity. However, this study is not without limitations. The cross-sectional design limits the ability to establish causality, as it only provides a snapshot of associations at a single point in time. Data collection via an online survey excludes individuals without internet access, potentially biasing the sample. Additionally, the generalizability of the results may be affected by demographic factors, such as the overrepresentation of females and the relatively young age of most participants. We also didn’t account for external factors which may contribute to the causality of symptoms such as: dietary habits, smoking and stress. Moreover, the reliance on non-objective diagnostic methods, such as the RSI and the SFAR, may compromise the accuracy of the conclusions. This may introduce subjectivity and the risk of overestimation, as well as recall bias. The use of self-reported tools was chosen for their practicality, cost-effectiveness, and non-invasive nature, enabling the collection of data from a large population.This limitation is particularly relevant given the limited availability of diagnostic resources in Sudan, where research and healthcare are underfunded. Additionally, a lack of local scientific literature in regard to the prevalence, morbidity, and mortality of the conditions studied further challenges the contextualization of our findings.

### Recommendations

To enhance the robustness and applicability of future research, several recommendations can be made: Future studies should consider the use of a longitudinal design to better establish causal relationships. Moreover, researchers should utilize alternative data collection methods to reach individuals without Internet access. For example, using face-to-face interviews or community outreach programs to capture a more representative sample and minimize selection bias. Also, efforts should be made to ensure a more diverse demographic representation in future studies. This includes recruiting participants from various states in Sudan, particularly those that are currently safe and have stable Internet access; this would upgrade the generalizability of the findings. Future research should prioritize the use of objective diagnostic methods alongside self-reported measures. Collaborating with healthcare professionals to obtain clinical confirmations would help mitigate issues related to subjectivity and recall bias. Furthermore, advocating for increased funding and resources for biomedical research in Sudan is crucial. Supporting local researchers and healthcare initiatives can help overcome existing barriers and improve the quality of research outcomes. Future studies should also consider the broader healthcare context in Sudan, including the availability of diagnostic resources. Moreover, replication of this study in other countries and age groups can enhance the scientific pool for our findings.Understanding these limitations can inform more effective research designs and interpretations of findings.

## Conclusion

We identified a strong association between AR and LPR. Individuals diagnosed with AR are six times more likely to report LPR symptoms. Despite a small sample of asthmatic patients, the findings suggest that asthma may affect the AR-LPR relationship through complex and overlapping mechanisms. Factors such as age, gender, socioeconomic status, and environmental allergens were significant, with younger adults showing higher LPR prevalence and females reporting a higher percentage of both AR and LPR symptoms. Genetic predisposition and inflammatory processes were suggested as potential mechanisms.

Addressing the reciprocal relationships between AR, LPR, and asthma can significantly enhance the quality of life for affected individuals, particularly in resource-limited settings like Sudan. Integrated care strategies and continued research are crucial steps toward achieving better patient outcomes and advancing healthcare in similar contexts.

## Electronic supplementary material

Below is the link to the electronic supplementary material.

Supplementary Material 1

## Data Availability

The data used and/or analysed during the current study are available from corresponding author upon reasonable request.
