# Supplementary material for: Investigating allergic rhinitis effects on laryngopharyngeal reflux in Sudanese people during the Sudanese armed conflict
Source: Sci Rep. 2025 Jul 2;15:23443. doi: 10.1038/s41598-025-07517-1 (PMC12222783; doi:10.1038/s41598-025-07517-1)
Supplement: Supplementary file 1 — Supplementary Material 1 [file 41598_2025_7517_MOESM1_ESM.pdf]

Questionnaire Arabic and Translated Version

#Arabic version:

المعلومات الشخصية:

-الجنس

1.ذكر

2.انثى

-العمر:...........(سنة)

-الإقامة الحالية:

1. الخرطوم
2. الجزيرة
3. النيل الأبيض
4. النيل الأزرق
5. نهر النيل
6. الشمالية
7. سنار
8. كسلا
9. البحر الأحمر
10. شمال كردفان
11. غرب كردفان
12. جنوب كردفان
13. شرق دارفور
14. غرب دارفور
15. جنوب دارفور
16. وسط دارفور
17. شمال دارفور
18. القضارف

- الوضع العائلي الحالي؟

1. مخطوب
2. متزوج
3. مطلق
4. عازب
5. أرمل

-هل تسجر سجارة او شيشية؟

1.نعم

2.لا

-المستوى التعليمي:

1. دكتوراه
2. ماجستير
3. بكالوريوس
4. ثانوي
5. أساس
6. غير متعلم

-السنة الدراسية اذا كنت طالبا جامعيا:

1. المستوى الأول
2. المستوى الثاني
3. المستوى الثالث
4. المستوى الرابع
5. المستوى الخامس
6. المستوى السادس
7. لست طالب جامعي

-الكلية التي تدرس بها:

1. طب بشري
2. صيدلة
3. طب أسنان
4. هندسة
5. تقانة معلومات
6. تمريض
7. تربية
8. قانون
9. علوم
10. آداب
11. اقتصاد ومحاسبة
12. إدارة أعمال
13. أخرى

-هل تعمل؟

1.نعم

2.لا

- هل الدخل العائلي؟

1. يكفي للأمور الرفاهية والكماليات غير الأساسية

2. يكفي لحاجات العائلة وبعض الكماليات الإضافية
3. يكفي لحاجات العائلة فقط
4. غير كافي أبداً لحاجات العائلة

المعلومات الطبية والتاريخ المرضي:

-هل تم تشخصيك طبياً بالأزمة؟

1.نعم

2.لا

-هل هنالك عضو في العائلة بحساسية في الأنف, الصدر أو الجلد؟

1.نعم

2.لا

-اذا نعم, ما هي نوع حساسية الأب:

1.حساسية الأنف

2.حساسية الجلد

3.حساسية الصدر

4.ليس لديه حساسية

-اذا نعم, ما هي نوع حساسية الأم:

1.حساسية الأنف

2.حساسية الجلد

3.حساسية الصدر

4.ليس لديها حساسية

-اذا نعم, ما هي نوع حساسية الأبناء:

1.حساسية الأنف

2.حساسية الجلد

3.حساسية الصدر

4.ليس لديهم حساسية

2.لا

-اذا نعم, ما هي نوع حساسية الاخوان:

1.حساسية الأنف

2.حساسية الجلد

3.حساسية الصدر

4.ليس لديهم حساسية

التاريخ المرضي للحساسية

-خلال السنة الماضية بغض النظر عن نزلات البرد والزكام (الرشح) هل عانيت من واحد أو أكثر من المشاكل التالية؟ (يمكن اختيار أكثر من إجابة)

1.سيلان الانف

2.احتقان الانف

3.العطاس

4.لم أعان

-في حال اخترت أي من الإجابات السابقة، خلال السنة الماضية هل كانت الحكة وتدميع العين مصاحبة لمشاكل الأنف السابقة؟

1.نعم

2.لا

-في أي من شهور السنة الماضية أو من فصول السنة حدثت مشكلة الأنف؟ (يمكن اختيار أكثر من إجابة)

1. شهر 1 يناير
2. شهر 2 فبراير
3. شهر 3 مارس
4. شهر 4 أبريل
5. شهر 5 مايو
6. شهر 6 يونيو
7. شهر 7 يوليو
8. شهر 8 أغسطس
9. شهر 9 سبتمبر
10. شهر 10 أكتوبر
11. شهر 11 نوفمبر
12. شهر 12 ديسمبر
13. لم تحصل لي

-ماهي العوامل التي قد تحفز من زيادة المشاكل الأنفية لديك؟ (يمكن اختيار أكثر من إجابة)

1.غبار المنزل

2.حشرات غبار المنزل

3.حبوب اللقاح من الشجر

4.البرد

5.التغيير في الجو

6.الحيوانات

7.العطور

8.اخرى

-هل تعتقد أنك تعاني من الحساسية؟

1.نعم

2.لا

-هل سبق وإن أجريت اختباراً للحساسية في الجلد أو الدم؟

1.نعم

2.لا

-ماهي نتيجة التحليل في حال قمت به؟

1. إيجابي، توجد حساسية
2. سلبي، لاتوجد حساسية
3. لم أقم به

-هل تم تشخصيك من قبل طبيب بحساسية في الصدر أو الأنف أو بحساسية جلدية؟

1.نعم

2.لا

مقياس أعراض الارتجاع

-خلال الشهر الماضي، بغض النظر عن نزلات البرد والزكام، بمقياس من 0 إلى 5 اختر درجة تأثير هذه المشاكل عليك مع العلم أن 0 تعني مشكلة صغيرة و 5 تعني مشكلة مؤثرة بشدة

تغير أو بحة في الصوت

إفرازات زائدة بالحنجرة أو من خلف الأنف

تنظيف الحنجرة (تقشع من الحنجرة)

صعوبة في بلع الأكل أو السوائل أو الأدوية

سعال بعد الأكل أو أثناء الاستلقاء أو النوم

صعوبة تنفس\شردقة

نوبة سعال مزعج شديد

إحساس بوجود جسم غريب عالق بالحنجرة

حرقان بفم المعدة، ألم بالصدر، عسرة هضم، صعود حموضة المعدة إلى الأعلى

#Translated version for publication:

Socio-demographic:

-Gender:

1.Male

2.Female

-Age:…….. (Years)

-Current residency:

1. River Nile
2. Kassala
3. North State
4. Red Sea
5. Khartoum
6. White Nile
7. Gazira
8. Al Qadarif
9. North Kordofan
10. Sennar
11. Blue Nile
12. West Kordofan
13. North Darfur
14. South Darfur
15. South Kordofan
16. East Darfur

-Marital status:

1. Single
2. Married
3. Engaged

4. Widow
5. Divorced

-Do you smoke cigarette or hookah:

1.Yes

2.No

-Educational level:

1. Illiterate
2. informal education
3. Primary School
4. Secondary school
5. Bachelor degree
6. Postgraduates

-Current grade if in university

1. 1st year
2. 2nd year
3. 3rd year
4. 4th year
5. 5th year
6. 6th year
7. Not currently a university student

-The faculty you are studying/have studied in:

1. Medical laboratory sciences
2. Public health
3. Nursing
4. Pharmacy
5. Dentistry
6. Medicine
7. Engineering
8. Information technology
9. Education
10. Law
11. Science
12. Literature
13. Economy and accounting
14. Business
15. Other

-Do you work?

1.Yes

2.No

-Is family income sufficient?

1. Not enough at all
2. Sufficient for basic needs only
3. Sufficient for basic needs and some additions
4. Sufficient for way above basic needs and luxuries

Clinical characteristics:

-Had you been clinically diagnosed by asthma?

1.Yes

2.No

-Do you have a member of the family with a nose, chest or skin allergy?

1.Yes

2.No

-If yes, what type is your father allergy (choose all that apply):

1. Nose allergy
2. Skin allergy
3. Chest allergy
4. Doesn't have allergy

-If yes, what type is your mother allergy (choose all that apply):

1. Nose allergy
2. Skin allergy
3. Chest allergy
4. Doesn't have allergy

-If yes, what type is your son/daughter allergy (choose all that apply):

1. Nose allergy
2. Skin allergy
3. Chest allergy
4. Doesn't have allergy

-If yes, what type is your siblings allergy (choose all that apply):

1. Nose allergy
2. Skin allergy
3. Chest allergy
4. Doesn’t have allergy

Allergies history:

-Regardless of having cold, did you suffer from any of the following during previous year (choose all that apply):

1. Running nose
2. Nasal congestion
3. Sneezing

-If you suffered from above mentioned symptoms, was coughing and lacrimation accompanying them?

1.Yes

2.No

-In what month of the year, the previous incident occurred (choose all that apply) ?

1. January
2. February
3. March
4. April
5. May
6. June
7. July
8. August
9. September
10. October
11. November
12. December

-Exacerbating Factors of allergy (choose all that apply):

1. Dust
2. Cold
3. Perfumes
4. Insects
5. Change in weather
6. Animals
7. Pollen

8. Others

-Do you believe you have an allergy?

1.Yes

2.No

-Have you took any skin or blood test for allergy?

1.Yes

2.No

-If yes, what was the test result?

1. I didn't have the test
2. Negative, there is no allergy
3. Positive, there is allergy

-Do you have diagnosis of skin, nose or chest allergy?

1.Yes

2.No

Reflex symptom index

Within the last month, how did the following problems affect you? 0=no problem, 5=severe problem
Circle the appropriate response.

1. Hoarseness or a problem with your voice
2. Clearing your throat
3. Excess throat mucus or postnasal drip
4. Difficulty swallowing food, liquids, or pills
5. Coughing after you ate or after lying down
6. Breathing difficulties or choking episodes
7. Troublesome or annoying cough
8. Sensations of something sticking in your throat or a lump in your throat
9. Heartburn, chest pain, indigestion, or stomach acid coming up
